# Health workers’ education and training to prevent antimicrobial resistance

**DOI:** 10.2471/BLT.19.241802

**Published:** 2019-12-01

**Authors:** Onyema Ajuebor, Nandini Shetty, Karen Mah, Giorgio Cometto

**Affiliations:** a Health Workforce Department, World Health Organization, avenue Appia 20, 1211 Geneva 27, Switzerland.; b National Infection Service Laboratories, Public Health England, London, England.; c Antimicrobial Resistance Department, World Health Organization, Geneva, Switzerland.

Antimicrobial resistance is a global public health concern that threatens to reverse the advancements achieved in medicine and health-care delivery. Competent and motivated health workers are essential to fight antimicrobial resistance. However, gaps in the education and training of health workers are curbing their ability to mitigate the spread of antimicrobial resistance.^1^ One such gap is the lack of global standards in education and training on this topic.^2^

At levels of health systems leadership and governance, challenges include lack of adequate policies on the appropriate use of medicines and poor implementation and monitoring of existing national or subnational-level medicines supply chains.^3^ These challenges often combine with other systemic factors that further entrench the irrational use of antimicrobials. 

Since the World Health Assembly’s adoption of the Global Action Plan on Antimicrobial Resistance in 2015,^4^ significant commitments in this direction, such as the Declaration of Astana,^5^ have followed. The renewed commitment to strengthen primary health care as a crucial driver to achieve universal health coverage (UHC) is a vital opportunity to tackle antimicrobial resistance at the base level of health systems. Strengthening the competencies of primary health-care workers on antimicrobial resistance can help countries achieve UHC and several sustainable development goals.

Several tools for competency-based education and training on antimicrobial resistance exist.^1^ The development of educational materials is centred on a stepwise process of establishing competency frameworks for health workers according to their roles and allowed scopes of practice, and then defining relevant and context-specific curricula, learning materials and assessment methods. This approach allows flexibility for health education institutions, regulators and health-care providers to determine their needs, local priorities and standards. The approach includes linkages to broader elements such as digital learning technologies and behavioural change strategies that contribute to a more responsible use of antimicrobials.

The first objective of the global action plan is to improve the awareness and understanding of antimicrobial resistance through effective communication, education and training of health workers. The World Health Organization (WHO) developed the global competency framework for education and training on antimicrobial resistance^6^ as a complementary global resource to help achieve this objective. 

The framework offers guidance on vital antimicrobial resistance-related competencies that are relevant for health workers across the spectrum of health-care delivery, including doctors, nurses, pharmacists, laboratory technicians, health services managers and public health officers, but also those in supportive or administrative roles. The framework also comprises core and additional competencies for health workers to address antimicrobial resistance in policy and practice settings in the field of human health. 

The competency framework includes the following areas: awareness of antimicrobial resistance, appropriate use of antimicrobial agents, infection prevention and control, and diagnostic stewardship and surveillance. The framework is mainly targeted at learners at pre-service and in-service health education and training institutions, and can be used in several ways. Uses include identifying needed antimicrobial resistance competencies that address local gaps, developing relevant teaching materials, and planning for the auditing and optimization of health workers skills to manage or mitigate antimicrobial resistance.

Building on this competency framework, WHO has also recently published a curricula guide^7^ for health workers education and training on antimicrobial resistance. The concept of antimicrobial stewardship as a coherent set of actions that contribute to ensuring the responsible use of antimicrobials^8^ should be mainstreamed as an overarching objective when developing antimicrobial resistance competency frameworks and curricula.

To address antimicrobial resistance, health workers will benefit from more and improved training on the subject, including good antimicrobial prescribing practice for clinicians.^9^ Strengthening in-service education and training is also vital to ensure the maintenance of competencies across all levels and roles. Linking local adaptation approaches with relevant broader contextual elements is encouraged. For instance, regarding methods of delivering education and training on antimicrobial resistance, evidence shows that digital learning platforms can improve antibiotics management, especially for in-service training.^10^ The growing number of subscribers to content on this topic indicates that such platforms are in popular demand.^11^

The use of the competency framework and curricula guide will help institutions and educators to adapt their antimicrobial resistance educational offer according to local needs and to develop relevant learning materials that can enhance efforts to combat antimicrobial resistance.
